# Assessment of hepatic fat content and prediction of myocardial fibrosis in athletes by using proton density fat fraction sequence

**DOI:** 10.1007/s11547-022-01571-9

**Published:** 2023-01-10

**Authors:** Tao Liu, Ping Dong, Jin-Rong Zhou, Jing Chen, Qian-Feng Luo, Shuang Long, Jia-Li Li, Dong Chen, Yuan-Sheng Li

**Affiliations:** 1 Department of Radiology, The Affiliated Hospital of Southwest Medical University, 25# Tai Ping Street, Luzhou, 646000 Sichuan China; 2 School of Public Health, Southwest Medical University, No.1, Section 1, Xianglin Road, Longmatan District, Luzhou, 646000 Sichuan China

**Keywords:** Liver fat content, Exercise, MRI-PDFF, Late gadolinium enhancement, Risk factor

## Abstract

**Purpose:**

To explore the characteristics of the hepatic fat content in athletes, and predict late gadolinium enhancement (LGE) based on magnetic resonance imaging-proton density fat fraction (MRI-PDFF).

**Material and methods:**

From March 2020 to March 2021, 233 amateur athletes and 42 healthy sedentary controls were prospectively recruited. The liver fat content of four regions of interest (ROIs 1–4), the mean liver fat fraction (FF), cardiac function, and myocardium LGE were recorded, respectively. The values of ROIs 1–4 and FF were compared between athletes and controls. According to the liver fat content threshold for distinguishing athletes and controls, the cutoff total exercise time that induced a change in liver fat was obtained. The correlations among the liver fat content, cardiac function, and other parameters were analyzed. Moreover, the liver fat content was used to predict myocardium LGE by logistic regression.

**Results:**

There were significant differences for the values of ROI 1, ROI 3, ROI 4, and FF between athletes and controls (all*p*< 0.05). The cutoff total exercise time for inducing a change in the liver fat content was 1680 h (area under the curve [AUC] = 0.593, specificity = 83.3,*p*< 0.05). Blood indexes, cardiac function, and basic clinical parameters were related to liver fat content (all*p*< 0.05). The prediction model for LGE had an AUC value of 0.829 for the receiver operator characteristic curve.

**Conclusion:**

MRI-PDFF could assess liver fat content and predict cardiac fibrosis in athletes for risk stratification and follow-up.

## Introduction

Moderate exercise intensity and duration can improve physical fitness. For example, exercise training can reduce liver fat accumulation and cardiovascular risk in patients with non-alcoholic fatty liver disease (NAFLD) [1]. Although findings highlighted the long-term beneficial effects of well-controlled exercise interventions on liver fat content and metabolic risk [2], excessive exercise could lead to cardiac damage such as myocardial fibrosis, which presents as a “U” shape in the correlation between exercise and cardiovascular health [3]. As the heart and liver are closely connected with each other [4], change in liver fat content is an early indicator of some liver diseases and is also important for body metabolism. How the liver fat content responds to exercise in terms of body health or cardiovascular events remains unclear. As reported, there was a negative relationship between obesity, diabetes, and N-terminal prohormone of brain natriuretic peptide/brain natriuretic peptide, which are robust biomarkers of subclinical left ventricular dysfunction and heart failure [5]. Whether the reduction in liver fat content is beneficial or not, similar to what occurs in the cardiovascular system by exercise, is uncertain. So we need to assess the characteristics of liver fat content in athletes.

About the liver fat’s response to exercise, most studies primarily focused on short-term clinical trials with supervised intervention. For example, one study demonstrated that walking for 60 min on a treadmill for 7 days could reduce liver fat in 15 obese people with NAFLD [6]. However, these articles did not conclude how long exercise accumulation might lead to the reduction of liver fat in exercisers or healthy people, and whether a certain amount of reduced liver fat content could be harmful to the body or cardiovascular system. In addition, there is no relevant research on the changes of liver fat content in long-term exercisers.

Liver biopsy remains the gold standard for evaluating liver fat content; however, its clinical applications are limited due to its harmful effects and sampling accuracy [7]. Magnetic resonance spectroscopy (MRS) is the most common method of measuring proton density fat fraction (PDFF) [8]. However, the availability of MRS is limited by poor measurement repeatability, and the interest frame can only be measured within certain areas of interest during scanning. Researchers need relevant, professional knowledge to successfully obtain and interpret results. Moreover, the price of needed machinery and equipment is expensive [9]. While, magnetic resonance imaging (MRI)-PDFF can accurately and repeatably quantitatively evaluate the liver fat for the whole liver, and is less expensive than MRS, making it suitable for replacing liver biopsy to evaluate the changes of liver fat content in the experiment [10].

At present, there are few studies on the change of liver fat content in long-term exercisers using MRI-PDFF. Therefore, this study aimed to explore the benefits and risks to the liver and heart based on the reduced liver fat content caused by exercise. Moreover, the model to predict late gadolinium enhancement (LGE) is constructed based on the liver fat content, as LGE indicates cardiac fibrosis in athletes and can be a proxy for cardiovascular adverse events [11]. Determining the cutoff time of exercises and predicting the future occurrence of LGE may provide useful suggestions for subsequent exercise programs.

## Materials and methods

### Study population

This study was approved by the Institutional Review Board of our hospital (Ref: KY2020123) in accordance with the Declaration of Helsinki (2013). All participants signed informed consent voluntarily and knowingly.

We prospectively recruited 233 amateur athletes from May 2020 to March 2021.The inclusion criteria were as follows: (1) exercise duration ≥ 3 years and exercise time ≥ 6 h/week, or meeting a total exercise time ≥ 864 h (with complete sports records and athlete certifications); (2) a demonstrated medical history of generally positive health, including the absence of liver disease, such as a fatty liver or systemic influence of liver disease, hyperthyroidism, diabetes, congenital heart disease, hypertrophic cardiomyopathy, and valvular disease; and (3) no history of metal implants, claustrophobia, or other MRI contraindications. The exclusion criteria were as follows: (1) stopping exercising for more than half a year; (2) any cardiovascular risk factors; (3) poor MRI image quality; and (4) taking specific drugs, such as muscle building drugs, within the past 1 year. Finally, 193 exercisers (172 men and 21 women; mean age, 25.92 ± 7.25 years; range, 17–57 years) remained in our study. In addition, 18 age- and sex-matched healthy sedentary volunteers (15 men and 3 women; mean age, 26.89 ± 7.79 years; range, 21–54 years) with normal examination results and no clinical symptoms were collected as the control group. And the control group exercised no more than 3 h per week and had no regular cumulative exercise time.

All participants were informed of prohibiting coffee consumption and strenuous exercise within 24 h and fasting for at least 4 h before the scan. They underwent a series of preliminary screening procedures, including height and weight assessments, detailed medical histories inquiry, routine electrocardiograms and static blood pressure measurements to rule out potential disease, and were informed of the procedures, purposes, and possible risks for the MRI examinations. Concurrently, of all the participants, 91 amateur athletes and 10 controls voluntarily underwent blood tests, including alanine aminotransferase (ALT), aspartate aminotransferase (AST), creatinine, uric acid, total cholesterol (TC), low-density lipoprotein cholesterol (LDL-c), high-density lipoprotein cholesterol (HDL-c), red blood cell specific volume (HCT), fasting plasma glucose, and serum TC.

### MRI protocol

All the participants underwent 3.0 T MRI scan (Siemens Healthineers, Prisma, Eriangen, Germany) with a body coil of 18 channels. The sequence and parameters of MRI-PDFF liver scanning were as follows: (1) Positioning images: True fast imaging with steady precession (true-FISP) was used to prospectively conduct conventional positioning images in transverse, sagittal, and coronal positions. (2) The imaging parameters were Fov = 450 mm × 393 mm, resolution = 160 × 112, TR = 9.00 ms, TE = 1.05 ms, flip angle = 4.0°, average = 1, slice thickness = 3.0 mm, slice spacing = 0.6 mm, and bandwidth = 1040.

The cardiac cine scanning used true-FISP sequences for two cavities, three cavities, four cavities, and 8–12-layer continuous short axis images of cine scan. The scanning parameters were as follows: TR = 66.2 ms, TE = 1.46 ms, layer thickness = 6 mm, layer spacing = 1 mm, and vision 340 × 270 mm^2^.

In addition, cardiac delayed-enhanced scanning was voluntarily performed 10 min after intravenous injection of 0.1 mmol/kg and 3 ml/s gadolinium. The scanning parameters were TR = 929.4 ms, TE = 1.24 ms, layer thickness = 6 mm, layer spacing = 1.2 mm, field of vision 340 × 270 mm^2^, and a 256 × 186 matrix.

### Image analysis

**Fig. 1:**
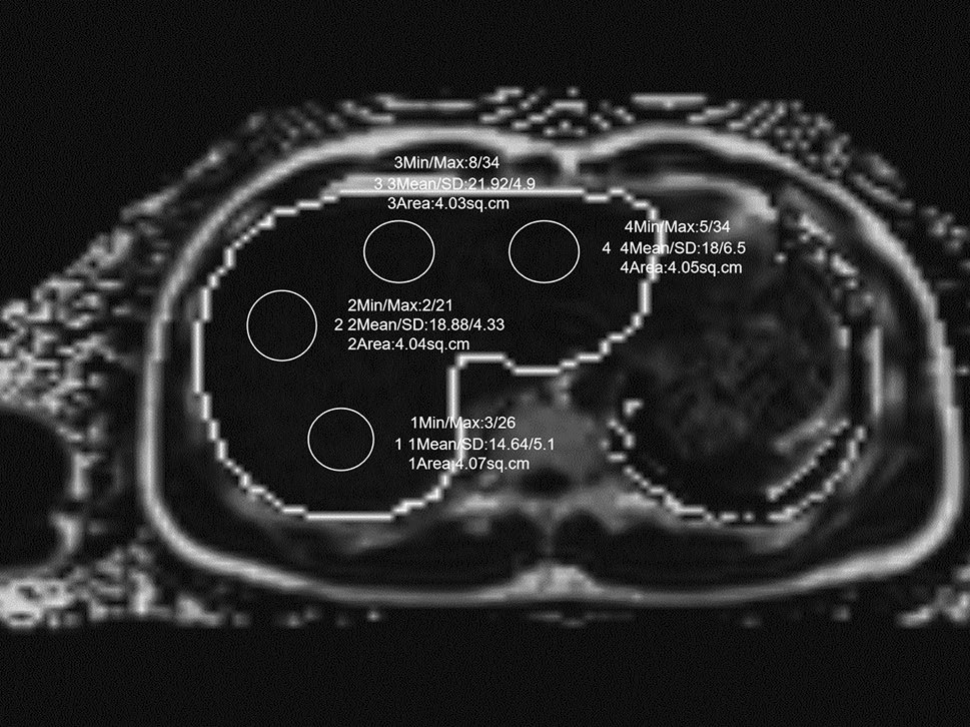
Schematic diagram for measurement of the liver fat content in MRI-PDFF image. The four ROIs (white circles) were outlined in the liver area (white curve), including the anterior, posterior, medial, and lateral areas, and the fat content was produced automatically. The average liver fat content of the four ROIs was taken as the average fat content of the whole liver

All liver fat content maps of amateur athletes were transmitted and determined in the post-processing workstation (SyngoVia, Siemens Healthineers, Erlangen, Germany). Based on the relevant study [7], use of the ROI 4 (≥ 4 cm^2^) paradigm (anterior, posterior, medial, and lateral) was preferred in this study to provide the ROI’s liver fat content. The liver fat content was calculated automatically by the processing workstation software. The liver was divided into four ROIs while avoiding large blood vessels, bile ducts, liver damage and image artifacts, as shown in Fig.1. Two-independent double-blinded radiologists with more than three years of experience in MRI imaging diagnosis measured them. The average liver fat content of the four ROIs was taken as the average fat content of the whole liver. In addition, all the above liver fat contents were corrected by the body mass index (BMI).

All cardiac data were transformed to CVI (Release 5.12.4, Circle Vascular Imaging, Calgary, Canada), a post-processing workstation for processing. By dragging the movie sequence images into the corresponding processing module, the software semiautomatically identifies the endocardium and epicardium of the left and right ventricles on the short axis. The above radiologists checked the contour for accuracy and manually sketched the endocardium and epicardium. The endocardium sketch did not include blood pool or papillary muscles, and the software automatically calculated the structural and functional parameters of the left ventricle (LV). These parameters mainly included end-diastolic volume (EDV), end-systolic volume (ESV), ejection fraction (EF), cardiac output (CO), cardiac index (CI), stroke volume (SV), ventricular mass, other cardiac function parameters, and body surface area (BSA) correction parameters.

The two-independent experienced radiologists simultaneously scanned the LGE images for high signals in the myocardium and determined if there were abnormal areas of enhancement. If there were any differences, they negotiated until a consensus was reached.

To determine the intra-observer variability, 30 volunteers were randomly selected from all the research participants and a PDFF image measurement was later made for a second time one month later by the same radiologist.

### Statistical analysis

All data were analyzed using the statistical software R (R 4.1.2 Core Team 2020, Vienna, Austria). Continuous variables were expressed as mean (standard deviation) and categorical variables were expressed as numbers. A rank-sum test was used to compare the differences in fat content and the basic clinical information between the control group and the amateur athlete group. We divided the athlete group into two subgroups according to the difference threshold of the average liver fat content for distinguishing athletes and controls. Then, we determined the exercise time threshold for inducing a change in fatty content by exercise using the receiver operator characteristic (ROC) analysis. Pearson’s correlation test was utilized to evaluate the relationship among the liver fat content, cardiac function, blood index and basic clinical parameters. Logistic regression analysis was used to screen variables of baseline data and liver fat content parameters for the outcome variable of LGE, which used the Akaike information criterion as a stopping rule in both the forward and backward directions. The screened variables were used to construct a prediction model for multivariate logistic regression, and the variance inflation factor (VIF) statistic was used to assess multicollinearity between the independent variables. VIF < 10 was calculated to exclude multicollinearity. The area under the ROC curve (AUROC) was used to measure the performance of the model in predicting LGE. Statistical tests were two-tailed, and*p*< 0.05 was considered to indicate statistical significance. A nomogram was built by these variables, and a Hosmer–Lemeshow (H–L) goodness-of-fit test was used to test the model fit. Intra- and inter-observer variability for reproducibility was evaluated using intraclass correlation coefficients (ICCs), where ICC > 0.8 represented high repeatability.

## Results

### Basic clinical data

**Fig. 2:**
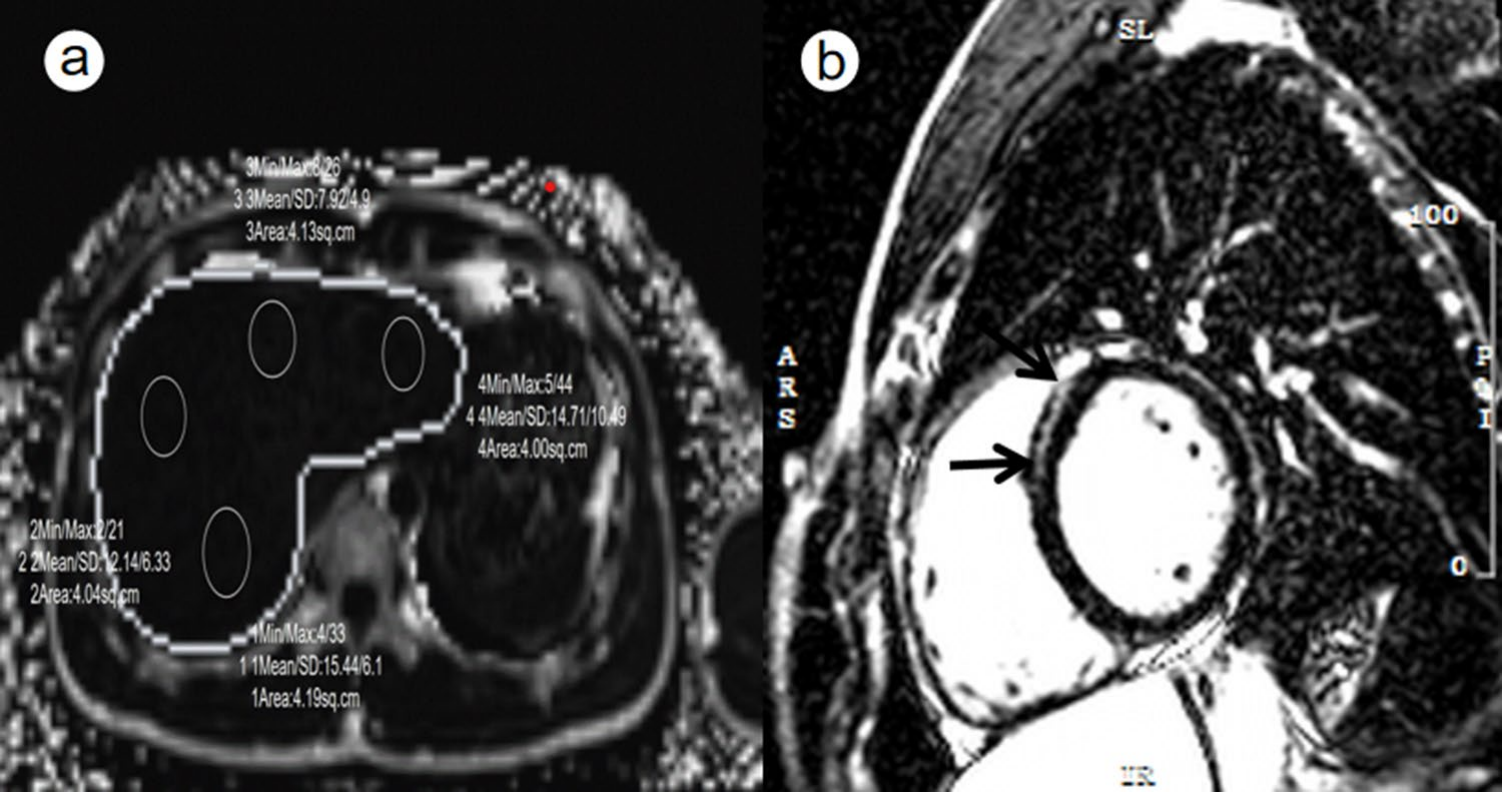
MRI images in a 26-year-old male athletes.The average liver fat content in MRI-PDFF image is 1.25% (the measured values of the ROIs 1–4 were 1.5%, 1.2%,0.8% and 1.5%, respectively)(Figure a, circle). Short axis images of late gadolinium enhancement depict a striped high signal in the middle myocardium of septal wall for left ventricle (Figure b, arrow)

The basic characteristics of the amateur athletes and the controls are shown in Table1.The average weekly exercise time in the amateur athlete group was 10.9 h, and the average years of exercise was 6.43 years. LGE was found in seven male amateur athletes, which mainly located in the middle myocardium of the left ventricular septum and inferior wall (Fig.2). The amateur athlete group had a lower resting cardiac rate than the control group (*p*< 0.05). There was no significant difference in blood test results between the athletes and the controls (*p*> 0.05).

**Table 1:** Basic clinical data of the healthy control and amateur athletes

Clinical data	Amateur athletes (*n*= 193)	Healthy controls (*n*= 18)	*P*-value
Age (years)	25.92 ± 7.25	26.89 ± 7.79	0.214
Gender (Male/Female)	172/21	15/3	0.655
Height (cm)	171.73 ± 6.51	170.39 ± 6.04	0.451
Weight (kg)	67.84 ± 9.53	65.61 ± 9.75	0.376
BSA (M^3^)	1.92 ± 0.15	1.84 ± 0.14	0.366
BMI (kg/m^2^)	22.97 ± 2.72	22.6 ± 3.2	0.681
SBP (mm/Hg)	115.82 ± 13.96	119.22 ± 12.6	0.877
DPB (mm/Hg)	76.03 ± 10.48	76.17 ± 11.82	0.968
Cardiac rate (/min)	67.3 ± 10.6	79.17 ± 10.61	**< 0.001**
Exercise time (H/week)	10.9	–	–

Blood index data	Amateur athletes (*n*= 91)	Healthy controls (*n*= 10)	*P*-value
ALT (U/L)	22.50 ± 16	17 ± 5.35	0.342
AST (U/L)	22.36 ± 8.77	17.84 ± 2.75	0.068
UA(cm)	374.22 ± 73.75	394.18 ± 95.26	0.577
Creatinine (μmol/L)	78.12 ± 10.58	81.87 ± 13.5	0.453
TC(mmol/L)	4.12 ± 0.76	4.33 ± 0.95	0.554
TG(mmol/L)	1.02 ± 0.51	0.95 ± 0.22	0.909
HDL (mg/dl)	1.37 ± 0.36	1.54 ± 0.24	0.058
LDL (mg/dl)	2.3 ± 0.58	2.57 ± 0.88	0.406
Fasting glucose (mg/dl)	4.73 ± 0.32	4.6 ± 0.23	0.171

Bold values are considered statistically significant

*BMI* body mass index; *BSA* body surface area; *SBP* systolic blood pressure; *DPB* diastolic blood pressure; *BMI* body mass index; *BSA* body surface area; *ALT* alanine aminotransferase; *AST* aspartate aminotransferase; *UA* uric acid; *TC* total cholesterol; *TG* triacylglycerol; *HDL* high density lipoprotein; *LDL* low density lipoprotein; *HCT* red blood cell specific volume

### Exercise cutoff time for inducing changes in liver fat content by exercise

There were significant differences in the fat content of ROI 1, ROI 3, ROI 4, and FF between the athletes and controls (*p*< 0.05). According to ROC analysis, the optimal cutoff value of mean liver fat content for distinguishing amateur athletes and controls was 1.016 (AUC = 0.703, specificity = 66.7). All athletes were grouped into two subgroups by this mean liver fat cutoff point, and there were significant differences in total exercise time between these two groups of athletes (*p*< 0.05). According to the ROC analysis, the optimal cutoff of total exercise time for the change in the liver fat content by exercise was 1680 h (AUC = 0.593, specificity = 83.3).

### Correlation between liver fat content and other parameters in amateur athletes

We analyzed the correlation among the liver fat content, cardiac function, and blood index of amateur athletes (Table2). We found that the liver fat content negatively correlated with some cardiac function parameters, including left ventricular EDV/BSA, right ventricular EDV/BSA and heart rate (*p*< 0.05). FF had a negative relationship with left cardiac EDV/BSA (*r*= −0.2873,*p*< 0.05). The value of ROI 2 had a negative relationship with right cardiac EDV/BSA (*r*= −0.229,*p*< 0.05). The value of ROI 4 had a positive relationship with heart rate (*r*= 0.2046,*p*< 0.05). BMI had a positive relationship with total exercise time and liver fat content (*r*= 0.156,*p*= 0.0432; and*r*= 0.418,*p*< 0.0001, respectively).

**Table 2:** The correlation among cardiac function, blood index and liver fat content of amateur athletes

		FF1	FF2	FF3	FF4	FF
RV EDV/BSA	*r*	− 0.2276	− 0.229	− 0.156	− 0.1487	− 0.2119
	*p*	0.0029	0.0028	0.0428	0.0536	0.0057
RV SV/BSA	*r*	− 0.2058	− 0.2165	− 0.1092	− 0.147	− 0.1914
	*p*	0.0073	0.0047	0.1576	0.0565	0.0127
RV ESV/BSA	*r*	− 0.158	− 0.1633	− 0.1255	− 0.1001	− 0.1477
	*p*	0.0401	0.0339	0.104	0.1954	0.0553
LV ESV	*r*	− 0.1535	− 0.1569	− 0.1333	− 0.1542	− 0.1691
	*p*	0.0463	0.0416	0.0839	0.0453	0.0279
HR	*r*	0.2205	0.159	0.1647	0.2046	0.2005
	*p*	0.004	0.039	0.0323	0.0076	0.009
LV EDV/BSA	*r*	− 0.2873	− 0.2593	− 0.2077	− 0.2405	− 0.2748
	*p*	0.0002	0.0007	0.0067	0.0016	0.0003
LV ESV/H	*r*	− 0.1567	− 0.1574	− 0.135	− 0.1461	− 0.1678
	*p*	0.0419	0.041	0.08	0.0581	0.0292
LV ESV/BSA	*r*	− 0.2476	− 0.2478	− 0.1773	− 0.2117	− 0.249
	*p*	0.0012	0.0012	0.0211	0.0057	0.0011
ALT	*r*	0.4335	0.409	0.3805	0.4607	0.4547
	*p*	< 0.0001	0.0001	0.0003	< 0.0001	< 0.0001
TG	*r*	0.3555	0.3617	0.3663	0.463	0.4044
	*p*	0.0007	0.0006	0.0005	< 0.0001	< 0.0001
LDL-c	*r*	0.2104	0.2957	0.2619	0.2988	0.2771
	*p*	0.0505	0.0054	0.0143	0.0049	0.0094
HDL-c	*r*	− 0.2692	− 0.2077	− 0.2675	− 0.2037	− 0.2492
	*p*	0.0117	0.0536	0.0123	0.0585	0.0199
AST	*r*	0.2138	0.1675	0.2005	0.207	0.216
	*p*	0.0467	0.1209	0.0626	0.0544	0.0445

Bold values are considered statistically significant

*FF1* the liver fat content of ROI 1; *FF2* the liver fat content of ROI 2; *FF3* the liver fat content of ROI 3; *FF4* the liver fat content of ROI 4; *FF* the mean liver fat fraction; *LV* left ventricle; *RV* right ventricle; *EDV* end diastolic volume; *ESV* End systolic volume; *HR* heart rate; *BSA* body surface area; *ALT* alanine aminotransferase; *TG* total cholesterol; *LDL-c* low-density lipoprotein cholesterol; *HDL-c* high-density lipoprotein cholesterol; *AST* aspartate aminotransferase

For the 91 athletes, the results of blood tests and the association between ALT, TG, LDL-c, HDL-c and FF are shown in Table2.ALT had a positive relationship with FF (*r*= 0.4545,*p*< 0.0001). FF had a positive and negative relationship with LDL-c and HDL-c levels (*r*= 0.2771,*p*< 0.05; and*r*= −0.2492,*p*< 0.05, respectively). The association of TG with left cardiac parameters are shown in Table3. TG had a negative relationship with left ventricular EDV/BSA (*r*= −0.2336,*p*< 0.05). BMI had a negative relationship with HDL-c (*r*= −0.169,*p*< 0.05).

**Table 3:** The correlation among cardiac function and TG of amateur athletes

Cardiac function	TG	
	*r*	*p*
HR	0.2177	0.0428
LV EDV/BSA	− 0.2336	0.0295
LV ESV/BSA	− 0.2207	0.04
LV Myo Mass/BSA (Syst)	− 0.2854	0.0074
LV Mass/BSA (Syst)	− 0.2516	0.0187

Bold values are considered statistically significant

*TG* total cholesterol; *LV* left ventricle; *HR* heart rate; *EDV* end diastolic volume; *ESV* end systolic volume; *BSA* body surface area; *Mass* myocardial mass

### Construction of a prediction model for LGE based on liver fat content

**Fig. 3:**
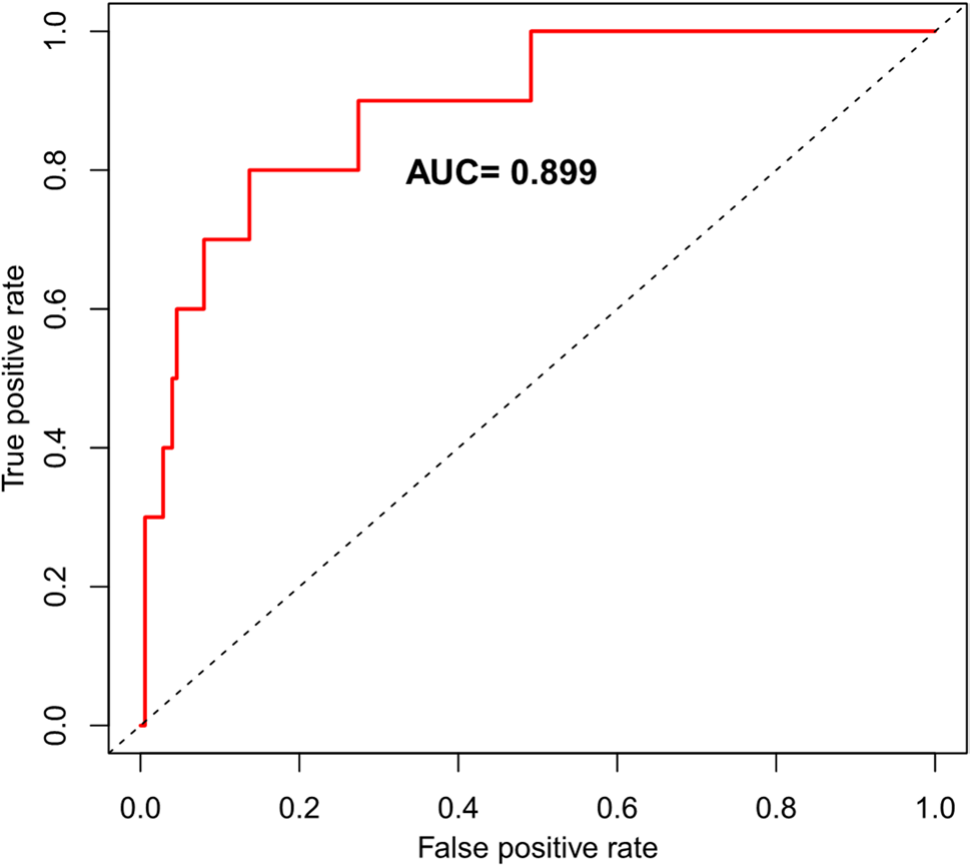
Receiver operator characteristic curves of the prediction model for myocardial late gadolinium enhancement. The area under the curve (AUC) was 0.899

**Fig. 4:**
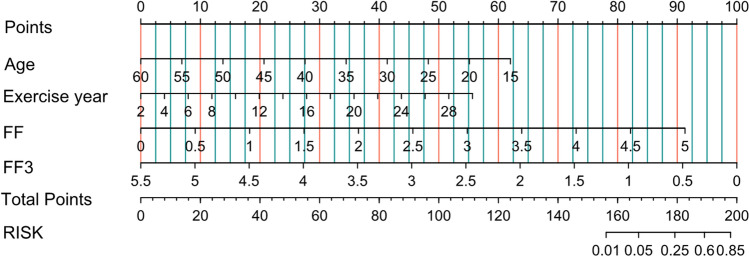
Nomogram of the prediction risk for myocardial late gadolinium enhancement (LGE). A line was drawn vertically from the corresponding axis of each risk factor until reaching the top row, which was labeled "point". Adding the scores of all risk factors and drawing a line down the axis marked "total point" until the risk axis is intercepted to determine the LGE probability. (FF,the average fat content of the whole liver; FF3, the liver fat content of ROI 3)

Due to the absence of myocardium LGE in female athletes, we excluded the small number of female athletes for the prediction model, so as to avoid the influence of sex. The logistic regression results are shown in Table4. The risk probability for the outcome of LGE was approximately 1% to 85%, and the AUROC curve of the model was 0.829 (Fig.3). In addition, there were four risk factors used in the model, including age, years of exercise, FF value, and ROI 3 in the nomogram (Fig.4).To illustrate the nomogram, for a 33-year-old amateur athlete with regular and frequent physical exercise with 18 years of sports history, a fat value of ROI 3 of 2.2, and FF value of 3.6, the total score would be calculated as 37 + 32 + 60 + 65 = 194. As such, this athlete has an approximately 80% probability of LGE. Using the H–L goodness of fit test, the model calibrated well. The*p*-value of goodness-of-fit was 0.9186.

**Table 4:** Logistic regression results of LGE prediction model

Characteristic	OR	95% CI	*p*-value
Year	0.81	0.59, 1.03	0.13
Exercise year	1.35	1.06, 1.74	**0.011**
FF	15.9	2.84, 192	**0.008**
FF3	0.06	0.00, 0.68	0.052
OR = Odds ratio, CI = Confidence interval			

Bold values are considered statistically significant

*LGE* late gadolinium enhancement; *FF* the mean liver fat fraction; *FF3* the liver fat content of ROI 3

### Inter-observer and intra-observer variability

The ICCs of intra-observer variability for the measurement of the above liver fat contents were 0.896–0.905, while the ICCs of inter-observer variability for the measurement of the above liver fat contents were 0.812–0.922.

## Discussion

### Main findings

In this study, we used MRI-PDFF to assess the liver fat content and predict LGE in athletes. Our study has several unique features. First, we obtained the length of exercise time that caused the liver fat content decreased via exercise. Second, our results also indicated that liver function and cardiac function parameters were related to liver fat content in amateur athletes, which indicates that liver and heart are closely related to each other. In view of the U-shaped relationship between exercise and the heart [3], we explored the relationship between the decrease in liver fat content and the myocardial fibrosis. Last, to the best of our knowledge, few studies have constructed a predictive model for LGE in amateur athletes with risk factors based on liver fatty content and other clinical data. Given that LGE is associated with cardiac fibrosis and represents adverse cardiovascular events [12, 13] for athletes, risk stratification may provide clinically relevant suggestions for athlete exercise plans.

### Characteristics of the liver fat content in athletes

Some studies have shown that exercise can independently interfere with liver fat without dietary restrictions, while BMI usually does not change [14]. Our results support the role of exercise as an independent intervention factor in fat content. About the recommended exercise program, any amount of exercise is beneficial to reduce the risk of development of new fatty liver or to improve the regression of existing fatty liver [15, 16], but the exercise time that could affect liver fat and the intensity of exercise for best managing high fatty liver content are uncertain. Moreover, there were large number of experiments on changing liver fat content by short-term exercise plans for patients with fatty liver or excessive deposition of liver fat content, and the amount of exercise is lower than the recommended exercise guidelines for the specific prevention and management of excessive weight and obesity [14]. However, we obtained a cutoff of exercise time to reduce the liver fat content in normal people, which was different and may provide suggestions for the exercise plan to reduce liver fat content for sports enthusiast.

AST and ALT are effective indicators of liver function and liver injury [17], and are often used in the diagnosis and treatment of liver diseases. Excessive triglyceride deposition, lipid peroxidation, and peroxide formation in the liver cause liver inflammation. The increase of inflammatory factors is an important pathophysiological mechanism of hepatocyte injury and destruction, which leads to changes in the serum liver enzyme level [17, 18]. Some studies have shown that serum ALT and AST levels in obese people are positively correlated with liver fat deposition [17, 18]. Our results are consistent with these previous studies. For the relationship between liver fat content and cardiac function, some studies have shown that excessive liver fat deposition is related to left ventricular structural changes and diastolic dysfunction, mainly manifesting as an increase in the left ventricular mass index, left ventricular end-diastolic diameter, and left atrial volume index [19]. However, this is contrary to our study, which found low liver fat content was related to the remodeling of cardiac function. When the liver fat content is too low or too high, cardiac function may be impaired. This response shows that a liver fat content that is too low is bad for the health of the heart. Therefore, more exercise is not always better, and moderate exercise should be followed. Relevant studies have shown that the levels of serum AST, ALT, and liver fat are positively correlated with TG [18]. At the same time, serum liver enzyme levels are closely related to the visceral fat content and systemic fat rate [20]. This is consistent with our results, which shows that an increase in BMI was closely related to the impairment of liver function.

### The ability of MRI-PDFF to predict future cardiac fibrosis

Delayed myocardial enhancement represents fibrosis in athletes, which is related to poor prognosis [11–13]. The existence of myocardial fibrosis may be an important cause of sudden cardiac death in athletes; several early studies have reported that myocardial fibrosis was found in the cardiac pathological anatomy of sudden death athletes [11, 21]. Early animal studies suggested that myocardial fibrosis caused by strenuous exercise reactivated myocardial interstitial fibrosis, which could be effectively reversed by intervention [22]. Therefore, early detection and correct evaluation of athletes’ early myocardial injury is conducive to early clinical intervention and reduces the risk of sudden cardiac death. Some reports have found that myocardial edema and fibrosis increase with the severity of liver damage, which is related to the activation of the renin angiotensin aldosterone system and the sympathetic nervous system [23]. In addition, myocardial edema and fibrosis appear to have a direct effect on the heart by inducing cardiac inflammation and oxidative stress [24–26]. This effect is also closely related to the excessive accumulation of liver fat. Therefore, we used the liver fat content for predicting LGE, so as to screen for relevant factors as soon as possible.

FF is one predictor for LGE; the higher the fat content is in the liver, the more likely is the development of cardiac fibrosis. The reason for this is that excessive deposition of liver fat content can promote the production of procoagulant factors, pro-inflammatory factors, pro-oxidant molecules and regulatory factors of promoting fibrogenesis, so as to accelerate the formation process of cardiac fibrosis and even heart disease [1, 24, 27]. Regarding the predictive ability of ROI 3, the lower the fat content is in the liver, the more likely is the development of cardiac fibrosis. This is contrary to the FF, because the liver fat content of ROI 3 is mainly representative of the left medial lobe of the liver, which may be the most sensitive region to assess changes in fat content compared with other ROIs, as there are Sappey veins with extra blood supply in this region in addition to the other normal liver blood supply [28, 29]. When the lipid content in the blood of these reflux veins increases, it leads to localized fat deposition, with false lesions around the sickle ligament in this area due to the rich blood supply. This is why focal fat deposition is often seen in the left medial lobe of liver. In contrast, when excessive exercise leads to a decrease in lipid of the blood, a decrease in fat content in this area should occur first. When the fat content in the ROI 3 area began to decrease, the liver fat content in the other three areas did not change, resulting in a relatively high average fat content across the whole liver (the value of FF was relatively high). However, the amount of exercise at this time may induce the LGE outcome. Therefore, in this model, the decrease of FF and ROI 3 will yield different results. In addition, this result also signifies the importance of paying close attention to changes of fat content in the ROI 3.

Another predictor was age. It is well-known that age is a risk factor for a range of diseases, such as coronary artery disease, hypertension, diabetes, and fatty liver [30]. The risk of cardiovascular adverse events in young people is much lower than that in elderly people. Another predictor was the duration of exercise time. Excessive exercise can cause myocardial damage. At the same time, some studies have shown that the degree of myocardial fibrosis is related to the number of years of exercise and the number of completed competitions [13, 31]. A prospective cardiac magnetic resonance study of marathon athletes found that 12% of 102 marathon athletes had myocardial fibrosis, and its incidence was three times than that of the normal control group [32]. The longer the cumulative exercise time, the higher the probability of cardiovascular adverse events, which supports the U-shaped relationship between exercise and heart health [3]. By combining clinical data with the MRI-PDFF sequences, we can perform an individual risk analysis of the subjects, which is different from making a judgment based on a single examination. This prediction model is more reasonable and can combine the characteristics of different individuals to make the predicted results more credible.

## Limitations

First, we recruited a small number of female athletes with an absence of myocardium LGE. Therefore, only male athletes were used in the prediction model to avoid the influence of sex. In future studies, we will recruit more female amateur athletes to examine the possible effects of sex. Second, the AUROC of the cutoff of total exercise time obtained was relatively low, which may be due to the smaller numbers and different exercise types of our recruited athletes. We attempted to analyze the data by exercise type, but the results were poor and needed more athletes for analysis. Finally, the number of delayed reinforcements of outcome variables in the prediction model was small, as LGE is common in the heavy-load amateur athletes (such as triathlon athletes) with a longer cumulative time of exercise, whose total number was low in our study. This study was a preliminary study, and we will recruit more LGE athletes in the next step.

In conclusion, exercise could lead to a decrease in liver fat content, which was found to occur with exercise over a specific amount of time and may be related to the myocardium LGE. As a noninvasive and convenient scan method, MRI-PDFF could successfully assess liver fat content and predict myocardial LGE in athletes. This approach may assist with risk stratification and suggestion of the frequency of follow-up for exercisers.
